# Comparison of Peri-operative and Early Oncological Outcomes of Robot-Assisted vs. Open Salvage Lymph Node Dissection in Recurrent Prostate Cancer

**DOI:** 10.3389/fonc.2019.00781

**Published:** 2019-09-04

**Authors:** Gaëtan Devos, Tim Muilwijk, Yannic Raskin, Victor Calderon, Lisa Moris, Thomas Van den Broeck, Charlien Berghen, Gert De Meerleer, Maarten Albersen, Hendrik Van Poppel, Wouter Everaerts, Steven Joniau

**Affiliations:** ^1^Department of Urology, University Hospitals Leuven, Leuven, Belgium; ^2^Laboratory of Molecular Endocrinology, KU Leuven, Leuven, Belgium; ^3^Department of Radiation Oncology, University Hospitals Leuven, Leuven, Belgium

**Keywords:** prostate cancer, salvage lymph node dissection, lymph node recurrence, robot-assisted approach, open approach

## Abstract

**Introduction:** Salvage lymph node dissection (sLND) has been proposed as a treatment option for prostate cancer patients with lymph node (LN) recurrence following radical prostatectomy to delay or avoid palliative androgen deprivation therapy (ADT). Historically sLND has been performed using an open approach, with its associated morbidity. A limited number of studies have reported peri-operative outcomes following robot-assisted sLND. However, a direct comparison with the open approach has hitherto not yet been reported. This study investigates whether robot-assisted sLND is associated with better peri-operative outcomes compared to the open approach. Early oncological outcomes are also compared.

**Patients and methods:** In this retrospective study, clinical data were collected from 60 patients undergoing open sLND between 2010–2016 and 30 patients undergoing robot-assisted sLND between 2016 and 2018 at our tertiary referral center. The primary objective of the study was to compare peri-operative outcomes (length of stay, estimated blood loss, operative time, intra-operative, and postoperative complications) and LN yield between both procedures. As secondary objective early oncological outcome [biochemical recurrence-free survival (BRFS) and clinical recurrence-free survival (CRFS)] was compared. Variables of interest were compared using the chi-squared test (categorical variables), two sample *t*-test, and Mann-Whitney *U*-test (continuous variables). To compare BRFS and CRFS, Kaplan-Meier analysis, and log-rank tests were performed.

**Results:** Robotic sLND was associated with reduced blood loss (median 100 vs. 275cc; *p* < 0.0001) and shorter length of stay (median 2 vs. 7 days; *p* < 0.0001) compared to open sLND. Moreover, postoperative complications within 30 days after surgery were more prevalent in the open sLND group compared to the robotic group (41.6% vs. 20%, *p* = 0.04). No significant differences in LN yield (for each sLND template), BRFS, and CRFS were detected between both groups.

**Conclusion:** Robot-assisted sLND is associated with significantly reduced peri-operative morbidity compared to open sLND. No difference in LN yield, BRFS and CRFS was seen between both groups. Modern imaging techniques underestimate the tumor burden and therefore, the surgical sLND template should not be limited to the positive spots on pre-operative imaging.

## Introduction

Biochemical recurrence (BCR) after radical prostatectomy (RP) for clinically localized prostate cancer occurs in 15–40% of patients (1, 2). With the emergence of new imaging modalities, such as choline and PSMA PET/CT, more patients are diagnosed with recurrence confined to a limited number of lymph nodes (LN) (3–6). These patients have a better prognosis than those with skeletal or visceral recurrence (1, 7, 8). In clinical practice, these patients are mainly treated with androgen deprivation therapy (ADT) which is a palliative option aimed at delaying symptoms (9). Recently, salvage lymph node dissection (sLND) has been proposed as a therapeutic option in “node-only” recurrence in order to postpone life-long palliative ADT or to possibly improve cancer-specific survival in selected patients (10–12). Historically, this procedure is performed using an open approach, with its associated morbidity (1, 13). Currently, a limited number of studies have reported peri-operative outcomes following robot-assisted sLND (14–17). However, a direct comparison with the open approach has hitherto not yet been published.

In this retrospective study we compared the peri-operative outcomes between open and robot-assisted sLND in patients with node-only recurrence following RP for clinically localized prostate cancer. We also compared early oncological outcomes between both procedures.

## Materials and Methods

### Patient Population

After obtaining approval from the institutional ethical review board (internal number: S61342), we retrospectively collected clinical data from patients undergoing open or robot-assisted sLND between 2010 and 2018 at a single tertiary referral center. Inclusion criteria were biopsy-proven diagnosis of adenocarcinoma of the prostate, BCR following RP (defined as confirmed PSA >0.2 ng/ml), at least one positive LN on imaging at the time of BCR, and open or robot-assisted sLND. Exclusion criteria were external beam radiotherapy (EBRT), brachytherapy, or high intensity focused ultrasound (HIFU) as initial treatment; visible recurrence in the prostatectomy bed; or concomitant skeletal (M1b) or visceral (M1c) recurrence on conventional or molecular-based imaging (as detected by one of the following imaging techniques at time of BCR: bone scan, abdomino-pelvic computerized tomography, MRI, and/or PET/CT).

### Patient and Tumor Characteristics

The following data were collected: clinico-pathological disease characteristics at RP, adjuvant/salvage ADT, or radiotherapy (RT) prior to sLND, imaging technique used at time of BCR, site of positive imaging (pelvic, retroperitoneal, or both), number of positive lesions on imaging, PSA at sLND, extent of sLND (pelvic, retroperitoneal, or both), number of LN removed at final pathology, perioperative blood loss (in cc), operative time (in min), and length of hospital stay (in days). Operative time was measured from skin incision to skin closure. Blood loss was estimated by the amount of blood aspirated during the procedure and weighing the surgical gauzes. Pre-operative morbidity of the patients was estimated by the age-adjusted Charlson-comorbidity index (CCI) (18). The BMI and American Society of Anesthesiologists (ASA)-classification at time of sLND was retrieved from the pre-operative anesthesia consultation (19).

### Surgical Technique

**Figure 1:**
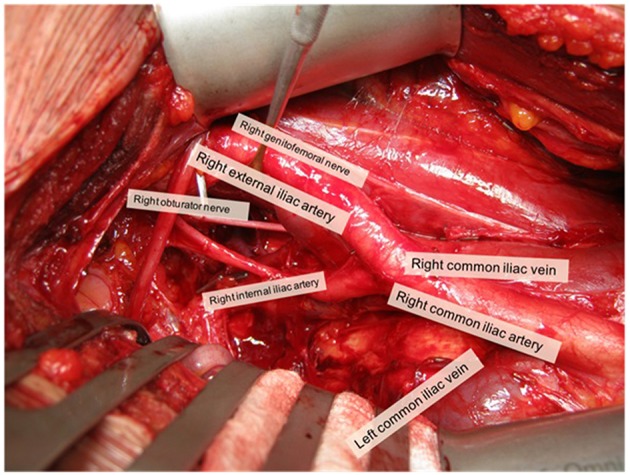
Overview of open pelvic sLND template (right side). Picture was taken with informed consent of the patient.

The pelvic sLND template was defined as the removal of LN distal to the aortic bifurcation (Figure 1) (20):

- *External iliac region:* tissue overlying the external iliac vessels. Borders: bifurcation of the common iliac vessels, circumflex iliac vein, psoas muscle, and genitofemoral nerve and medial border of the external iliac vein.
- *Obturator fossa region:* tissue lying below the iliac vessels and above the obturator nerve. Borders: bifurcation of the common iliac vessels, pelvic floor, obturator muscle, obturator nerve, and medial border external iliac vein.
- *Internal iliac region:* tissue lying around the internal iliac vessels. Borders: bifurcation of the common iliac vessels, pelvic floor, bladder wall, and obturator nerve.
- *Common iliac region:* tissue overlying the common iliac vessels. Borders: aortic bifurcation, bifurcation of the common iliac vessels, psoas muscle and genitofemoral nerve, and medial border of the common iliac vein.
- *Presacral region:* tissue overlying the proximal sacral bone. Borders: Triangle between medial borders of common iliac veins and the line connecting the bifurcations of the common iliac vessels; dorsal border: promontory and proximal sacrum (S1–S2).

**Figure 2:**
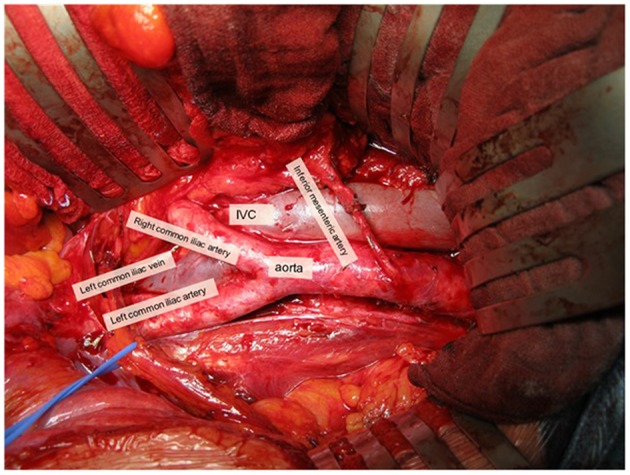
Overview of retroperitoneal sLND. Picture was taken with informed consent of the patient.

The retroperitoneal sLND template was defined as the removal of para-aortic and inter-aorto-caval LN above the aortic bifurcation up to the inferior mesenteric artery (or up to the renal hilum in case of nodal recurrence above the inferior mesenteric artery on pre-operative imaging) (Figure 2). Paracaval LN were only removed in case of a positive LN in that area on preoperative imaging. Templates were not limited to the positive spots on imaging and could be modified slightly according to the nodal recurrence site on pre-operative imaging and the extent of the prior pelvic LN dissection during RP.

All procedures were performed by three experienced surgeons (H.V.P., S.J., and W.E.). For the robot-assisted procedures, the Xi Surgical System (Intuitive Surgical, Sunnyvale, CA, USA) was used with a six-port transperitoneal approach. Supplementary Figure 1 provides an overview of port placement in pelvic sLND and retroperitoneal sLND. In the open sLND group, pelvic LN were approached by extraperitoneal access and retroperitoneal LN by transperitoneal access.

Preoperative bowel preparation was not performed. All patients received postoperative compression stockings and subcutaneous injections with low-molecular weight heparins.

### Primary Objective: Comparison of Perioperative Outcome

Intra-operative complications were retrieved from the surgical reports. Postoperative complications up to 30 days after sLND were retrieved by reviewing the electronic medical records and graded using the Clavien-Dindo classification (21). Complications later than 30 days postoperatively were not collected. Intra- and postoperative complications were reported according the recommendations of the European Association of Urology (EAU)-guidelines panel (22).

Lymph node yield for each type of sLND template (pelvic, retroperitoneal, or pelvic + retroperitoneal) was collected and compared between both approaches. Furthermore the proportion of positive LN on preoperative imaging/positive LN at final pathology was calculated and stratified by imaging technique (^11^C-choline vs. ^68^Ga PSMA-11 PET/CT) and surgical approach (open vs. robotic approach).

### Secondary Objective: Comparison of Early Oncological Outcome

Biochemical recurrence free-survival (BRFS) and clinical recurrence free-survival (CRFS) were compared between both groups. BCR was defined as a PSA-value >0.2 ng/ml post sLND and clinical recurrence was defined as the onset of new lesions on imaging (or if patients became symptomatic). Decisions on performing imaging following sLND was at the discretion of the treating physician and adjuvant/salvage treatments following sLND were decided at the multidisciplinary team meeting. Patients who did not have oncological follow-up data available were excluded from analysis (BRFS and CRFS).

### Statistical Analysis

Non-normally distributed continuous variables were reported by medians and interquartile ranges (IQRs) and normally distributed continuous variables by means and standard deviations (SDs). Summary statistics for categorical variables were reported using proportions and frequencies. Categorical variables were compared using the chi-squared test or Fisher's exact test and continuous variables using the two sample *t*-test or Mann-Whitney *U*-test. Kaplan-Meier analysis was performed to assess BRFS and CRFS, and log-rank test to determine a significant difference between both approaches. Statistical analyses were performed using the statistical software Medcalc, Statistical Software version 18.9 (MedCalc Software bvba, Ostend, Belgium; http://www.medcalc.org; 2018) with a significance level of *p* < 0.05.

## Results

### Baseline Patient Characteristics

Table 1 provides an overview of the baseline demographic and tumor characteristics according to surgical technique (open vs. robot-assisted) at time of RP. We identified 60 patients undergoing open sLND between 2010–2016 and 30 patients undergoing robotic sLND between 2016 and 2018. Patients in the open SLND group more often had Gleason score 8–10 prostate cancer compared to the robotic group (*p* = 0.03). No difference in proportion adjuvant/salvage radiotherapy was observed between the open and robotic groups [71.6 vs. 67.7%, respectively (*p* = 0.7)]. The (adjuvant/salvage) radiation field (mostly 66 Gy) was confined to the prostate bed. None of the patients were castration resistant at time of sLND. In total, 45 (75%) and 18 (60%) patients received a concomitant lymphadenectomy at time of RP in the open and robot group, respectively. Of these, information on the number of LN removed during RP was available in 34 (75.6%) and 12 (66.7%) patients in the open and robotic approach, respectively. No difference was observed in median number of LN removed during RP. Table 2 provides an overview of the baseline characteristics at time of sLND. No difference in preoperative morbidity was observed in terms of BMI, ASA-classification, and age adjusted CCI. In both groups the majority of the patients had oligometastatic recurrence defined as 1–3 lesions. At time of BCR, almost all patients (96.7%) in the robot-assisted group were assessed by ^68^Ga-PSMA-11 PET/CT compared to only 44% in the open group (*p* < 0.0001). More than half of these patients were evaluated by ^11^C-choline PET/CT (53.3%).

**Table 1:** Baseline characteristics at time of RP.

**Variable**	**Open sLND *n* = 60 (66.6%)**	**Robotic sLND *n* = 30 (33.3%)**	***p*-value (two-tailed)**
**Mean age at RP, years (SD)**	61.2 (6.9)	61.3 (6.4)	0.54
**pT-stage**			0.37
T2	20 (33.3%)	9 (30%)	
T3a	24 (40%)	8 (26.7%)	
T3b-4	15 (25%)	11 (36.7%)	
Tx	1 (1.6%)	2 (6.7%)	
**pN-stage**			0.64
N0	38 (63.3%)	16 (53.3%)	
N1	7 (11.6%)	3 (10%)	
Nx	15 (25%)	11 (36.6%)	
**Number of LN removed at RP, median (IQR)**	9 (5–18.5)	13.5 (5–19)	0.67
**pGleason**			**0.03**
6	1 (1.7%)	4 (13.4%)	
7	23 (38.3%)	16 (53.3%)	
8–10	31 (51.7%)	8 (26.7%)	
NA	5 (8.3%)	2 (6.7%)	
**Positive surgical margin**	23 (38.3%)	10 (33.3%)	0.68
**Post-RP treatment**			0.08
ADT only post-RP	6 (10%)	0	
RT only post-RP	29 (48.3%)	15 (50%)	
ADT + RT post-RP	14 (23.3%)	5 (16.7%)	
No post-RP treatment	9 (15%)	10 (33.3%)	

*Patients were stratified according to the surgical technique received (Open vs. robot assisted sLND). Data are presented as n (%) unless otherwise noted. ADT, androgen deprivation therapy; RT, radiation therapy; sLND, salvage lymphadenectomy; RP, radical prostatectomy; IQR, interquartile range; SD, standard deviation; NA, Not available. Bold p-values mean statistical significance*.

**Table 2:** Baseline characteristics at time of sLND.

**Variable**	**Open sLND *n* = 60 (66.6%)**	**Robotic sLND *n* = 30 (33.3%)**	***p*-value (two-tailed)**
**PSA (ng/ml) at sLND, median (IQR)**	1.6 (0.7–3.4)	1.1 (0.7–2.6)	0.25
**Mean age at sLND, years (SD)**	67.8 (6)	65.6 (5.5)	0.11
**BMI at sLND, median (IQR)**	26.3 (24.5–30.8)	26.05 (23.5–28.7)	0.26
**ASA classification at sLND**			0.65
1	5 (8.3%)	0	
2	39 (65%)	26 (86.7%)	
3	16 (26.7%)	4 (13.3%)	
**Age-adjusted CCI**			0.92
1	1 (1.7%)	0	
2	4 (6.8%)	3 (10%)	
3	25 (41.7%)	13 (43.3%)	
4	18 (30%)	8 (26.7%)	
5	8 (13.3%)	5 (16.7%)	
6	2 (3.4%)	1 (3.3%)	
7	2 (3.4%)	0	
**Type of imaging used**			**<0001**
11C-Choline PET/CT	32 (53.3%)	0	
68Ga-PSMA-11 PET/CT	25 (41.7%)	29 (96.7%)	
MRI	2 (3.4%)	1 (3.3%)	
CT	1 (1.7%)	0	
**Site of positive imaging**			0.36
Pelvic	47 (78.3%)	23 (76.7%)	
Retroperitoneal	8 (13.3%)	2 (6.7%)	
Both	5 (8.3%)	5 (16.7%)	
**Median number of positive lesions on imaging, (IQR)**	2 (1–3)	2 (1–2)	0.55
**Number of positive lesions on imaging**			0.81
1–3 lesions	52 (86.7%)	27 (90%)	
>3 lesions	8 (13.3%)	3 (10%)	

*Patients were stratified according to the surgical technique received (Open vs. robot assisted sLND). Data are presented as n (%) unless otherwise noted. PSA, prostate specific antigen; sLND, salvage lymphadenectomy; IQR, interquartile range; SD, standard deviation; BMI, body mass index [weight (kg)/length^2^ (m)]; ASA, American Society of Anesthesiologists (19); CCI, Charlson-Comorbidity Index. Bold p-values mean statistical significance*.

### Perioperative Outcomes

Table 3 provides an overview of the intra-operative and postoperative outcomes and complications. Patients treated with robot-assisted sLND had significantly less estimated blood loss during the procedure compared to open sLND (median 100 vs. 275cc; *p* < 0.0001). However, no intra-operative transfusions were needed in either group. Median operative time between the two procedures was equal (median 150 vs. 150 min; *p* = 0.89). Length of stay was significantly lower in the robot-assisted sLND group compared to the open sLND group (median 2 vs. 7 days; *p* < 0.0001). No difference in intra-operative complications was observed (*p* = 0.34), but postoperative complications within 30 days after surgery were significantly more prevalent in the open group compared to the robotic group (41.7 vs. 20%, *p* = 0.04). Moreover, patients in the open group had more high-grade complications [5 vs. 0 Clavien-Dindo grade III-IV complications; hydronephrosis (double-J stent), arterial bleeding (reoperation), lymphocoele drainage (2x), renal failure (biopsy was taken to exclude nephrological disease)]. Injury to the iliac veins was the most prevalent was the most prevalent intra-operative complication in both groups. Postoperatively, lymphatic complications were more prevalent in the open group.

**Table 3:** Peri-operative outcomes of patients treated with sLND according to type of procedure (open vs. robotic).

**Variable**	**Open sLND *n* = 60 (66.6%)**	**Robotic sLND *n* = 30 (33.3%)**	***p*-value (two-tailed)**
**Area sLND**			
Pelvic	37 (61.7%)	20 (66.7%)	0.79
Retroperitoneal	5 (8.3%)	3 (10%)	
Pelvic + retroperitoneal	18 (30%)	7 (23.3%)	
**Median operative time, min (IQR)**	150 (120–175)	150 (120–180)	0.89
**Median blood loss, ml (IQR)**	275 (175–675)	100 (25–162.5)	**<0.0001**
**Median length of stay, days (IQR)**	7 (6–10)	2 (2–3)	**<0.0001**
**Intraoperative complications**	13 (21.7%)	4 (13.3%)	0.34
Vascular injury (vein)	7	2	
Bladder perforation	2	0	
Ureteral lesion	1	0	
Vascular injury (artery)	1	1	
Nerve injury	1	0	
Chyle leakage	1	0	
Pressure wound left shoulder	0	1	
**Postoperative complications** ** <30 days after sLND (Clavien-Dindo classification)**	25 (41.7%)	6 (20%)	**0.04**
I-II	20	6	
III-V	5	0	
**Type postoperative complication**			
Lymphatic:	7	2	
*Symptomatic lymphocele*	3	0	
*Symptomatic scrotal edema*	3	0	
*Chyle leakage*	1	1	
*Symptomatic lymph oedema legs*	0	1	
Fever/infection	5	0	
Ileus	4	0	
Hydronephrosis	1	0	
Renal failure	1	0	
Stomach bleeding	2	0	
Pulmonary embolism	1	0	
Dyspnea	1	0	
Arterial bleeding	2	0	
Arrhythmia	1	0	
Symptomatic hematoma	0	1	
Pain/stiffness right leg	0	1	
Hyperglycemia	0	1	
Painful left scrotum	0	1	

*Data are given as n (%) unless otherwise noted. sLND, salvage lymphadenectomy; SD, standard deviation; IQR, interquartile range; LN, lymph nodes. Bold p-values mean statistical significance*.

Table 4 provides an overview of the pathological outcomes. The number of LN removed for each sLND template (pelvic, retroperitoneal, and pelvic + retroperitoneal) was equal for both groups (*p* = 0.88, *p* = 0.24, and *p* = 0.85, respectively). A total of 477 LN were positive at final pathology, whereas only 200 (41.9%) metastatic LN were detected on imaging. Mean numbers of metastatic LN at final pathology were 4.1 (95%-CI: 2.5–5.7) and 6 (95%-CI: 2.7–9.2) in patients assessed by ^11^C-choline and ^68^Ga-PSMA PET/CT, respectively (*p* = 0.30). ^11^C-Choline PET/CT was able to detect 57 (42.8%) out of the 133 and ^68^Ga-PSMA PET/CT to detect 134 (41.8%) out of 320 metastatic LN at final pathology. No significantly difference in number of metastatic LN at final pathology was observed between the open and robotic group (*p* = 0.11).

**Table 4:** Pathological outcomes of patients treated with sLND according to type of procedure (open vs. robotic).

**Variable**	**Open sLND *n* = 60 (66.6%)**	**Robotic sLND *n* = 30 (33.3%)**	***p*-value (two-tailed)**
**Number of LN removed at sLND**	17 (9–26)	15 (10–27)	0.88
**Number of LN removed/sLND template**			
Pelvic	16 (6.5–24.75)	15 (8.5–25.5)	0.88
Retroperitoneal	17 (10.75–23)	10.5 (10–11)	0.24
Pelvic + retroperitoneal	20 (10–26)	23 (10.25–33)	0.85
**Number of positive LN removed at sLND**	3 (1–7)	1 (1–3)	0.11

*Data are given as median (IQR) unless otherwise noted. LN, Lymph node; sLND, salvage lymphadenectomy*.

### Early Oncological Outcome

**Figure 3:**
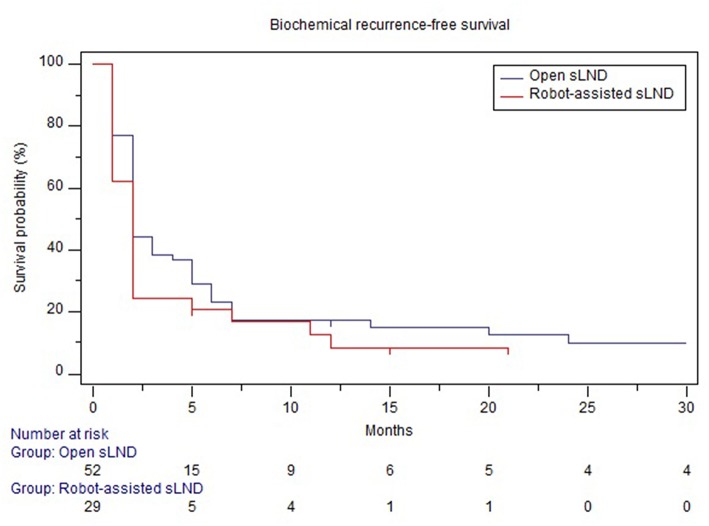
Comparison of biochemical recurrence-free survival between open and robotic sLND. Censored patients are marked with small vertical lines.

**Figure 4:**
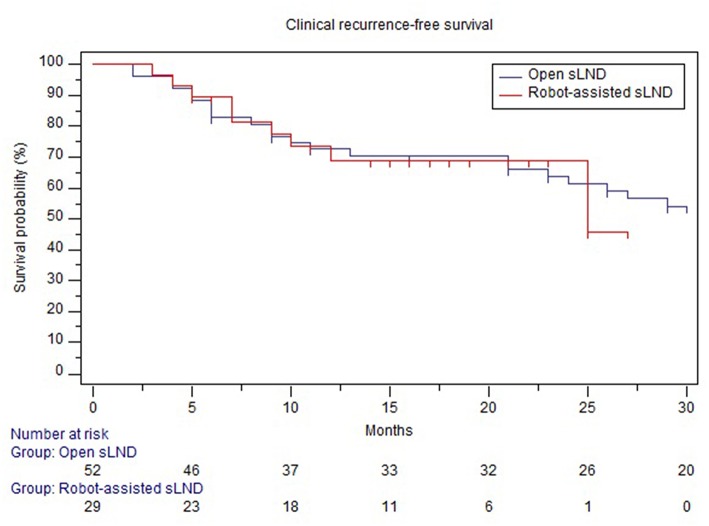
Comparison of clinical recurrence-free survival between open and robotic sLND. Censored patients are marked with small vertical lines.

Mean follow-up after open and robotic sLND was 53 (median: 53mo., IQR 31.5–75) and 15 (median 15mo., IQR 10.25–21.5) months, respectively (*p* < 0.001). Follow-up data were available for 52 (86.7%) patients in the open group and 28 (93.3%) patients in the robotic group. Supplementary Table 1 provides information on adjuvant/salvage therapies following sLND. In the open and robotic group, 38.4 and 58% of the patients received adjuvant or salvage treatment, respectively. Median BRFS was similar in both groups (2 months, *p* = 0.23) (Figure 3). The majority of patients in both groups experienced BCR (90 and 89%, respectively). No difference was observed in CRFS between both groups [median 25 mo. vs. 32 mo. in the robotic and open group, respectively (*p* = 0.87); Figure 4].

To correct for the difference in type of preoperative imaging between both groups, a sub-analysis of patients assessed by only ^68^Ga-PSMA-11 PET/CT was performed. Supplementary Table 2 provides an overview of the baseline demographic and tumor characteristics according to surgical technique (open vs. robot-assisted). Baseline tumor characteristics were balanced between both groups. No difference in BRFS (median 2 mo. in both groups, *p* = 0.59) and CRFS (median not attained in the open group and median of 25 months in the robotic group, *p* = 0.79) were observed between the open and robotic approach (Supplementary Figures 2, 3).

## Discussion

Patients with prostate cancer recurrence confined to a limited number of LN following primary treatment, also called oligometastatic recurrence, are potential candidates for metastasis-directed therapies. The EAU-guidelines introduced sLND as a possible therapeutic option in these patients. Salvage LND is typically performed by an open approach and associated is with substantial morbidity (1, 13). Four studies have so far investigated the feasibility and peri-operative outcomes of robot-assisted sLND, though no direct comparison has been made with the open procedure (14–17). The current study aimed to investigate the peri-operative and early oncological outcomes between open and robot-assisted sLND in patients with LN recurrence after RP.

Several observations of our study are interesting. First, robot-assisted sLND appears to be a safe alternative with favorable perioperative outcomes compared to the open approach. No high-grade postoperative complications were seen in the robotic group. This is in line with previously published robotic sLND series, where very few high-grade complications were reported (14–17). Only in the series of Linxweiler et al., five patients (13.9%) experienced high-grade (grade III according to Clavien-Dindo) complications (17). Notably, in our study lymphatic complications were more frequent in the open group (28% of all complications). This might be explained by the fact that the pelvic nodes were approached by an extraperitoneal access in the open sLND group (in case of pelvic sLND), while all nodes were removed via a transperitoneal approach in the robot-assisted group (23–25). Moreover, patients in the open group had higher metastatic burden at final pathology compared to the robotic group (median 3 vs. 1 metastatic LN). This might partially explain the higher proportion of intra- and post-operative complications as bulky nodal disease can be associated with increased risk of complications. Further, our results demonstrated significantly less blood loss and a 5-day shorter hospital stay in the robotic group compared to the open group. The higher proportion of postoperative complications with the open approach might explain this difference in hospital stay. Median operation time (150 min) and median blood loss (100 ml) in the robotic cohort were comparable with the previously published robotic sLND series (range 129–228 min and 50–250 ml, respectively) (14–17). Remarkably, the median operation time—generally one of the major drawbacks for robotic procedures—was not different between both groups. Also the number of LN removed for each sLND template (pelvic, retroperitoneal, and pelvic + retroperitoneal) was not different between both groups.

Second, only 200 (41.9%) out of 477 positive LN at final pathology were visible on preoperative imaging. Remarkably, ^68^Ga-PSMA-11 PET/CT was not superior to ^11^C-Choline PET/CT to identify metastatic LN. This might partly be explained by the fact that the mean metastatic burden in patients assessed by ^68^Ga-PSMA-11 PET/CT was higher compared to patients assessed by ^11^C-Choline PET/CT (although statistically not significant). Some patients who were assessed by ^68^Ga-PSMA-11 PET/CT had a very high proportion of positive LN at final pathology. For example, one patient had four suspect lesions on ^68^Ga-PSMA-11 PET/CT and received an open pelvic + retroperitoneal sLND resulting in 70 metastatic LN out of 78 at final pathology. Thus, despite the improved accuracy of novel imaging modalities at low PSA values compared to conventional imaging techniques, sLND should certainly not be limited to the positive spots on pre-operative imaging (26). Today, no consensus exists about the optimal extent of the sLND template. Therefore, radioguided surgery in which metastatic LN are detected intra-operatively with the use of a gamma probe, could provide an interesting alternative to reduce the morbidity of these (extensive) templates (27). Recently, Maurer et al. demonstrated that ^99^mTc-PSMA-based radioguided surgery had a good accuracy (93%) with promising early oncological outcomes in 31 patients with LN recurrence following RP (28). However, their technique still required an open approach with its associated morbidity (38.7% grade I; 3.2% grade IIIa complications). New promising technologies are currently developed that enable the use of radioguided surgery in combination with robotic surgery, leading to a further decrease in morbidity (29).

Finally, the majority of patients treated with sLND developed BCR independent of the surgical approach and in most cases BCR developed quickly (median time to BCR 2 months). As a consequence, it is important to counsel patients of the non-curative character of the procedure. Probably, CRFS rather than BRFS should be considered as a meaningful endpoint as CRFS in both groups extended 2 years. Patient selection appears to be of utmost importance for sLND. The identification of the “ideal” sLND candidate has already been investigated in a retrospective multi-center study in which our patients were included (30). Gleason grade group 5, a short time from RP to PSA rising, hormonal therapy at the time of sLND, positive retroperitoneal spots on imaging, ≥3 positive spots on PET scan and high PSA at time of sLND were significant predictors for early clinical recurrence (<1 year following sLND). These patients had a worse cancer-specific survival compared to patients who developed clinical recurrence >1 year following sLND. Similar prognostic factors were identified in patients treated by PSMA-based radioguided surgery (22). These findings underline the need for prospective studies to evaluate the oncological usefulness of sLND and to assess the added value of adjuvant treatments. Currently, a prospective phase-2 study (NCT03569241) is investigating the additional value of pelvic RT following sLND.

Our study is not devoid of limitations. First, this is a single center, retrospective case series comparing two techniques and is as such prone to several types of bias (31). Second, patient cohorts were not contemporary: half of the patients in the open group were assessed by ^11^C-choline PET/CT, whereas almost all patients in the robotic group were assessed by ^68^Ga-PSMA-11 PET/CT. This is important as ^11^C-choline PET/CT is less accurate at low PSA values than ^68^Ga-PSMA PET/CT (32–34). As a consequence, half of the patients in the open group might have been understaged (occult metastases) compared to their counterparts in the robotic group and more patients with local recurrence might have been missed by choline PET/CT and therefore (falsely) not excluded from the study, both resulting in a worse oncological outcome. Third, patients in the robot-assisted group had less aggressive tumor characteristics (less Gleason score 8–10 at final pathology following RP) and a shorter follow-up compared to their counterparts in the open group. Therefore, we cannot definitively conclude from this data that both sLND approaches provide similar early oncological outcomes. However, a sub-analysis of only those patients who received a PSMA PET/CT at time of BCR showed no difference in BRFS and CRFS.

Notwithstanding these limitations, the extent of surgical templates was identical with both techniques, as was the number of nodes removed within each of the templates (pelvic, retroperitoneal, and pelvic + retroperitoneal). Both groups had comparable baseline patient characteristics (e.g., no difference in post-RP adjuvant/salvage RT proportion between both groups). Moreover, no differences in terms of preoperative co-morbidities (age adjusted CCI, ASA, and BMI) were observed. We therefore believe that the conclusions on surgical feasibility, perioperative, and postoperative complications of this study are reliable. Moreover, this is the first series comparing intra-operative, postoperative and early oncological outcomes between open and robotic sLND.

## Conclusions

Robotic salvage lymph node dissection appears to be a safe alternative for the open procedure with the associated benefits of minimally invasive surgery, including shorter length of stay, lower estimated blood loss, and lower early postoperative complication rates. No difference in early BRFS and CRFS was seen between both groups. Modern imaging techniques underestimate the tumor burden and therefore, the surgical sLND template should not be limited to the positive spots on pre-operative imaging.

## Data Availability

The datasets generated for this study are available on request to the corresponding author.

## Ethics Statement

The studies involving human participants were reviewed and approved by Ethische commissie UZ Leuven. Written informed consent for participation was not required for this study in accordance with the national legislation and the institutional requirements. Written informed consent was obtained from the individual(s) for the publication of any potentially identifiable images or data included in this article.

## Author Contributions

GD and SJ: study concept and design. GD, TM, and YR: acquisition of data. GD: analysis and interpretation of data, drafting of the manuscript, and statistical analysis. VC, MA, CB, GM, LM, TV, HV, WE, and SJ: critical revision of the manuscript for important intellectual content. SJ: supervision.

### Conflict of Interest Statement

The authors declare that the research was conducted in the absence of any commercial or financial relationships that could be construed as a potential conflict of interest.

## Supplementary Material

Supplementary Table 1 Post-sLND treatments according to type of procedure (open vs. robotic).

Supplementary Table 2 Baseline characteristics of patients with lymph node recurrence after radical prostatectomy treated with open or robot-assisted sLND and who were assessed by PSMA PET/CT.

Supplementary Figure 1 **(A)** Overview of port placement in case of pelvic robot-assisted sLND. A six-port transperitoneal approach is used. The camera-port (8 mm) is placed supra-umbilical. The robotic ports are placed at the same height as the camera-port: two on the left and one on the right side (red dots, 8 mm trocars). A 12 mm assistant port is placed on the right side (green dot) and a 5 mm assistant port (blue dot) is placed 5 cm higher between the right robotic port and the camera-port. **(B)** Overview of port placement in case of pelvic+retroperitoneal robot-assisted sLND. Ports are placed 5 cm higher compared to port placement in pelvic sLND. Pictures were taken with informed consent of the patient.

Supplementary Figure 2 Comparison of biochemical recurrence-free survival between open and robotic sLND for patients who received a PSMA PET/CT. Censored patients are marked with small vertical lines.

Supplementary Figure 3 Comparison of clinical recurrence-free survival between open and robotic sLND for patients who received a PSMA PET/CT. Censored patients are marked with small vertical lines.
