# Supplementary material for: Comparison of Peri-operative and Early Oncological Outcomes of Robot-Assisted vs. Open Salvage Lymph Node Dissection in Recurrent Prostate Cancer
Source: Front Oncol. 2019 Sep 4;9:781. doi: 10.3389/fonc.2019.00781 (PMC6737006; doi:10.3389/fonc.2019.00781)
Supplement: Supplementary Table 1 — Post-sLND treatments according to type of procedure (open vs. robotic). [file Table_1.DOCX]

| Supplementary table 1: Post-sLND treatments according to type of procedure (open vs. robotic) | | |
| --- | --- | --- |
| Variable | **Open sLND**  **n=52** | **Robotic sLND**  **n=29** |
| All patients  ADT only  ADT+RT  RT only | 11 (21%)  8 (15.4%)  1 (1.9%) | 7 (24.1%)  10 (34.4%)  0 |
| Patients who developed clinical recurrence  ADT only  ADT+RT  RT only | 9 (17.3%)  2 (3.8%)  1 (1.9%) | 0  1 (3.4%)  0 |
| Patients who did not develop clinical recurrence  ADT only  ADT+RT  RT only | 2 (3.8%)  6 (11.5%)  0 | 7 (24.1%)  9 (31%)  0 |
| Data are given as n (%) unless otherwise noted. ADT= Androgen Deprivation therapy, RT= Radiotherapy. | | |
