# Supplementary material for: Comparison of Peri-operative and Early Oncological Outcomes of Robot-Assisted vs. Open Salvage Lymph Node Dissection in Recurrent Prostate Cancer
Source: Front Oncol. 2019 Sep 4;9:781. doi: 10.3389/fonc.2019.00781 (PMC6737006; doi:10.3389/fonc.2019.00781)
Supplement: Supplementary Table 2 — Baseline characteristics of patients with lymph node recurrence after radical prostatectomy treated with open or robot-assisted sLND and who were assessed by PSMA PET/CT. [file Table_2.DOCX]

| Supplementary table 2: Baseline characteristics of patients with lymph node recurrence after radical prostatectomy treated with open or robot-assisted sLND and who were assessed by PSMA PET/CT. | | | |
| --- | --- | --- | --- |
| Variable | **Open sLND**  **n=19** | **Robotic sLND**  **n=28** | **p-value**  **(two-tailed)** |
| Mean age at RP, years (SD) | 61.05 (7.1) | 60.5 (6.3) | 0.8 |
| pT-stage  T2  T3a  T3b-4  Tx | 8(42.1%)  7(36.8%)  4(21%)  0 | 9(32.1%)  7(25%)  11(39.3%)  1(3.5%) | 0.36 |
| pN-stage  N0  N1  Nx | 11(57.9%)  3(15.8%)  5(26.3%) | 14(50%)  3(10.7%)  11(39.3%) | 0.63 |
| pGleason  6  7  8-10  NA | 1(5.2%)  5(26.3%)  9(47.3%)  4(21%) | 4(14.3%)  15(53.6%)  8(28.6%)  1(3.5%) | 0.23 |
| Positive surgical margin | 6(31.6%) | 10(35.7%) | 0.86 |
| PSA (ng/ml) at sLND, median (IQR) | 2.4(0.7-3.3) | 1.0(0.7-2.4) | 0.25 |
| Mean age at sLND, years (SD) | 68.3(5.4) | 65.7(5.5) | 0.13 |
| Median follow-up post-sLND, months (IQR) | 26(13-33) | 15.5(9.5-21.5) | 0.006 |
| Data are presented as n (%) unless otherwise noted. ADT= androgen deprivation therapy; RT=radiation therapy; sLND=salvage lymphadenectomy; RP=radical prostatectomy; IQR= interquartile range; SD= standard deviation; NA= not available. | | | |
