# Supplementary figures and images for: Comparison of Peri-operative and Early Oncological Outcomes of Robot-Assisted vs. Open Salvage Lymph Node Dissection in Recurrent Prostate Cancer
Source: Front Oncol. 2019 Sep 4;9:781. doi: 10.3389/fonc.2019.00781 (PMC6737006; doi:10.3389/fonc.2019.00781)

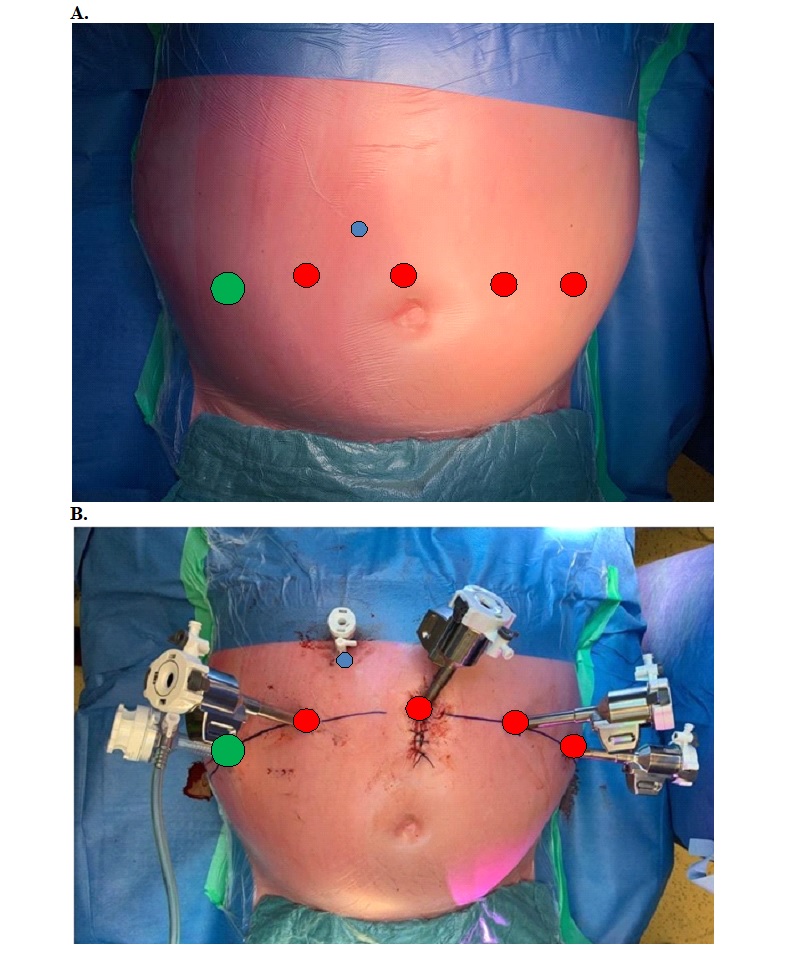

Supplement: Supplementary Figure 1 — (A) Overview of port placement in case of pelvic robot-assisted sLND. A six-port transperitoneal approach is used. The camera-port (8 mm) is placed supra-umbilical. The robotic ports are placed at the same height as the camera-port: two on the left and one on the right side (red dots, 8 mm trocars). A 12 mm assistant port is placed on the right side (green dot) and a 5 mm assistant port (blue dot) is placed 5 cm higher between the right robotic port and the camera-port. (B) Overview of port placement in case of pelvic+retroperitoneal robot-assisted sLND. Ports are placed 5 cm higher compared to port placement in pelvic sLND. Pictures were taken with informed consent of the patient. [file Image_1.JPEG]

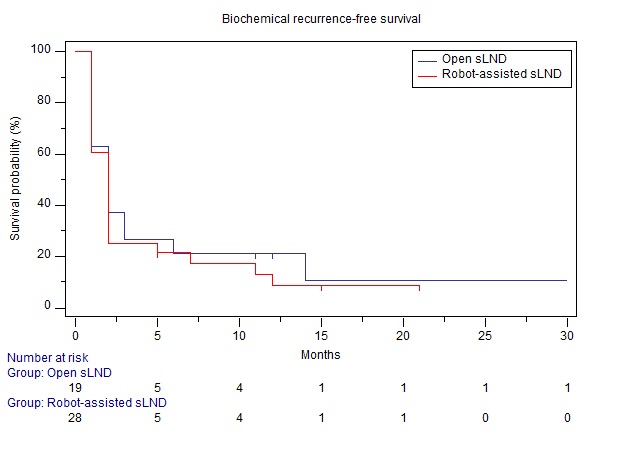

Supplement: Supplementary Figure 2 — Comparison of biochemical recurrence-free survival between open and robotic sLND for patients who received a PSMA PET/CT. Censored patients are marked with small vertical lines. [file Image_2.JPEG]

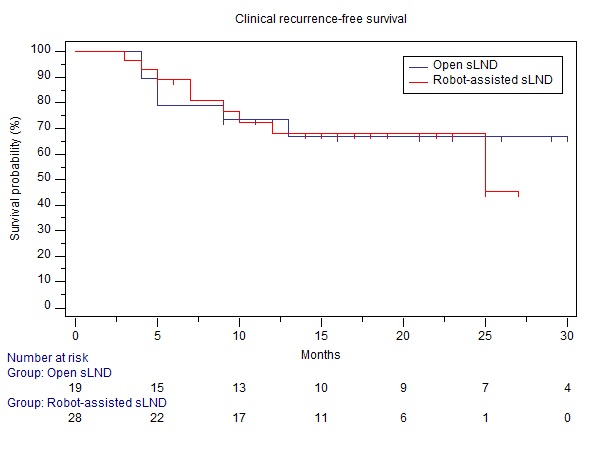

Supplement: Supplementary Figure 3 — Comparison of clinical recurrence-free survival between open and robotic sLND for patients who received a PSMA PET/CT. Censored patients are marked with small vertical lines. [file Image_3.JPEG]
